# Novel atypical G protein-coupled receptor (GPCR)-arrestin complexes: a structural snapshot of the barcode hypothesis

**DOI:** 10.1038/s41392-025-02338-0

**Published:** 2025-08-07

**Authors:** Jenny C. Filor, Edda S. F. Matthees, Carsten Hoffmann

**Affiliations:** Institut für Molekulare Zellbiologie, CMB – Center for Molecular Biomedicine; Universitätsklinikum Jena; Friedrich-Schiller-Universität Jena, Jena, Germany

**Keywords:** Structural biology, Structural biology

In a recent study published in *Nature* by Chen et al*.*, six novel cryo-EM structures of atypical chemokine receptor 3 (ACKR3) complexes with Arrestin2 (Arr2, also known as β-arrestin1) and Arrestin3 (Arr3, also known as β-arrestin2) were resolved using a novel nanobody, Fab7, which stabilizes active arrestin independent of the isoform, interacting receptor or its phosphorylation pattern.^1^ This work provides critical insights into G protein-coupled receptor (GPCR)–arrestin interactions under specific GPCR kinase (GRK) phosphorylation conditions, allowing an unprecedented direct comparison of these dynamic signaling complexes.

For the last decade, a central objective in structural biology has been to elucidate GPCR–arrestin complexes to better understand specific receptor regulation. One main challenge is to stabilize these naturally dynamic interactions for their structural illumination. The research teams of Tracy M. Handel and John J.G. Tesmer majorly advanced this endeavor with the development of their novel nanobody Fab7 as a powerful tool for future structural investigations. Crucially, this enabled the direct comparison of GRK2- or GRK5-specific receptor phosphorylation and its effect on GPCR–arrestin complex formation.^1^

**Fig. 1:**
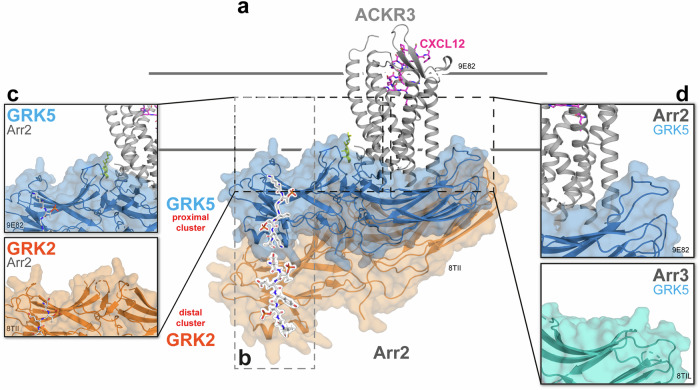
The dynamic conformational landscape of arrestins upon complex formation with ACKR3. **a** Cryo-EM structure of GRK5-phosphorylated ACKR3 with Arr2 (PDB: 9E82). The utilized ligand CXCL12_LRHQ_ is shown in pink and captured membrane lipids are indicated in yellow. The resolved Arr2 structure with the GRK2-phosphorylated ACKR3 C-terminus (orange, PDB: 8TII) was positioned to symbolize the register shift of interacting with the more distal phosphorylation cluster, leading to a more loosely associated complex and hence yielding a higher flexibility. **b** Differential C-terminal cluster phosphorylation by GRK5 (proximal, T338-T341) and GRK2 (distal, T352-S355), interacting with Arr2 (light gray box). **c** Close-up of Arr2 finger loop region (FLR), inserting into the micelle membrane upon GRK5-mediated phosphorylation, which could not be fully resolved in complex with the GRK2-phosphorylated ACKR3 C-terminus. **d** Close-up of the C-edge region of Arr2 (PDB: 9E82) and Arr3 (PDB: 8TIL) upon interacting with the GRK5-phosphorylated ACKR3

GRK-specific phosphorylation patterns, their impact on receptor–arrestin complexes and the associated biology are often summarized as the “barcode hypothesis”. More specifically, it states that unique phosphorylation patterns at the receptor C-terminus or intracellular loops, induced by the availability or activation of specific GRKs, may result in distinct receptor–arrestin complex formations and defines the arrestin-mediated functional outcomes.^2^ Now, Chen et al*.* provide long-awaited structural evidence for this concept.^1^ The comparison of different phosphorylation patterns by GRK5 (proximal) or GRK2 (distal) elucidate new details of the ACKR3–arrestin complex formations and reveal an additional degree of flexibility to arrestin biology (Fig. 1a). The authors demonstrate that the relative distance of the phosphorylated cluster on the receptor C-terminus in relation to the transmembrane (TM) core has a great influence on the orientation and stability of the resulting complex. The utilized ACKR3 is atypical in the sense that it does not activate G proteins but exhibits phosphorylation-dependent interactions with both Arr2 and Arr3. Cellular assays indicate that the ACKR3 is mainly regulated by GRK5,^3^ which localizes to the plasma membrane independently of active free G protein βγ-subunits as opposed to the cytosolic GRK2/3 subfamily.^4^ However, ACKR3 can also be phosphorylated by GRK2, following the release of Gβγ subunits by co-activation of the CXCR4 receptor with the shared ligand, if Gβγ dimers are abundantly expressed.^3^ This cross-regulatory mechanism suggests a scenario where ACKR3 can act as a sensor for CXCR4 activity and influence the efficiency of chemokine scavenging.^1^

The high-resolution cryo-EM structure of the GRK5-phosphorylated ACKR3–Arr2 complex (Fig. 1a, PDB: 9E82) reveals interaction details of both the phosphorylated C-terminal region (T338–T341) and the TM core of the receptor with arrestins. Under these conditions, Arr2 is drawn into a more tightly interacting complex with the receptor (Fig. 1a). In contrast, the more distal GRK2-phosphorylation pattern (PDB: 8Tll) at T352-S355 led to more flexibility of the overall complex and a more loosely associated arrestin (Fig. 1b). In both structures, the arrestin finger loop region (FLR) was shown to interact with the surrounding membrane rather than inserting into the active cavity of the receptor. Notably, the exact FLR position could not be resolved in the GRK2-phosphorylated state, highlighting its high conformational heterogeneity (Fig. 1c). Using fluorescence quenching assays, Chen et al*.* collected additional evidence, further supporting that the FLR can indeed interact with the micelle membrane for both isoforms in a way that resembles the glucagon receptor–arrestin complex.^1,2^ Interestingly, mutation of FLR revealed that for the GRK5-phosphorylated state, the binding affinity of Arr2 is less dependent on this specific interaction site. In addition, Chen et al*.* were able to distinguish arrestin isoform-specific interaction interfaces also at the C-edge region, after interaction with the same phosphorylation pattern of one GPCR (Fig. 1d). This region marks an important difference between the arrestin isoforms, as it is naturally shorter in Arr3, potentially adding to its increased flexibility and heterogeneity of formed receptor complexes.

In this study, the authors elegantly expanded the definition of arrestin binding requirements to the ACKR3 in relation to other observed phosphorylation motifs.^1,5^ By using an additional 12 amino acid Gly-linker to extend the distance between the GRK5-phosphorylated cluster and the receptor TM region, the resulting ACKR3–arrestin complex was shifted towards the GRK2-specific phenotype although phosphorylated via GRK5. Based on the GRK2 or GRK5-phosphorylated residues in the ACKR3 and the minor influence of the particular sequence but rather the localization of the interacting phospho-peptide, Chen et al*.* defined the ACKR3-specific phosphorylation barcode as *X*(pT)*XX*(pS/T)*X*Φ (where *X* represents any amino acid, while Φ symbolizes hydrophobic residues).^1^ In clear and focused analyses, they compared their findings to previous identified phosphorylation motifs with specific amino acid patterns and their occurrence in other GPCRs.^1,5^ Finally, the authors were able to integrate these results into a refined consensus sequence for arrestin interactions: requiring two closely located phosphorylated (or negatively charged) residues in combination with a hydrophobic anchor or third position phosphorylation (P*XX*PΦ).^1^

Chen et al*.* provide structural evidence to understand the impact of GRK-mediated phosphorylation patterns on arrestin-dependent outcomes.^1^ The formation of GPCR–arrestin complexes is determined by the proximity of these sites to the receptor’s TM core in addition to the specific phosphorylation “barcode”. This will also affect the additionally utilized stabilizing interaction interfaces, which might differ between the arrestin isoforms. Hence, it should be considered to broaden the “barcode hypothesis” to include a more holistic view of the structural determinants of GPCR–arrestin complexes. As the authors highlight, this insight is essential for understanding the pathophysiological regulation of GPCRs and advancing the rational design of arrestin-biased ligands, offering a more nuanced view of biased signaling that incorporates GRK-driven modulation.
